# Spatial and temporal variability of rotational, focal, and irregular activity: Practical implications for mapping of atrial fibrillation

**DOI:** 10.1111/jce.15170

**Published:** 2021-07-28

**Authors:** Michael TB Pope, Pawel Kuklik, Andre Briosa e Gala, Milena Leo, Michael Mahmoudi, John Paisey, Timothy R Betts

**Affiliations:** ^1^ Department of Cardiology Oxford University Hospitals NHS Foundation Trust Oxford UK; ^2^ Faculty of Medicine University of Southampton Southampton UK; ^3^ Department of Cardiology Asklepios Clinic St. Georg Hamburg Germany; ^4^ University of Oxford Biomedical Research Center Oxford UK

**Keywords:** AcQMap, atrial fibrillation, charge density mapping, localized irregular activation, spatiotemporal stability

## Abstract

**Background:**

Charge density mapping of atrial fibrillation (AF) reveals dynamic localized rotational activation (LRA), irregular activation (LIA) and focal firing (FF). Their spatial stability, conduction characteristics and the optimal duration of mapping required to reveal these phenomena and has not been explored.

**Methods:**

Bi‐atrial mapping of AF propagation was undertaken using AcQMap (Acutus Medical) and variability of activation patterns quantified up to a duration of 30 s. The frequency of each pattern was quantified at each unique point of the chamber over two separate 30‐s recordings before ablation and *R* ^2^ calculated to quantify spatial stability. Regions with the highest frequency were identified at increasing time durations and compared to the result over 30 s using Cohen's kappa. Properties of regions with the most stable patterns were assessed during sinus rhythm and extrastimulus pacing.

**Results:**

In 21 patients, 62 paired LA and RA maps were obtained. LIA was highly spatially stable with *R* ^2^ between maps of 0.83 (0.71–0.88) compared to 0.39 (0.24–0.57), and 0.64 (0.54–0.73) for LRA and FF, respectively. LIA was most temporally stable with a kappa of >0.8 reached by 12 s. LRA showed greatest variability with kappa >0.8 only after 22 s. Regions of LIA were of normal voltage amplitude (1.09 mv) but showed increased conduction heterogeneity during extrastimulus pacing (*p* = .0480).

**Conclusion:**

Irregular activation patterns characterized by changing wavefront direction are temporally and spatially stable in contrast with LRA that is transient with least spatial stability. Focal activation appears of intermediate stability. Regions of LIA show increased heterogeneity following extrastimulus pacing and may represent fixed anatomical substrate.

**Abbreviations**
ACT: activated clotting time
AF: atrial fibrillation
CHI: conduction Heterogeneity Index
FF: focal firing
LA: left atrium
LIA: localized irregular activation
LRA: localized rotational activation
MAT: median activation time
persAF: persistent atrial fibrillation
RA: right atrium

## 1 INTRODUCTION

The limited efficacy of pulmonary vein isolation for the ablation of persistent atrial fibrillation (persAF) has resulted in concerted efforts to identify nonpulmonary vein mechanisms responsible for AF maintenance. This has led to the development of techniques to facilitate mapping of the underlying atrial electrophysiology with the aim of revealing fibrillatory mechanisms and guiding targeted ablation.^1^, ^2^, ^3^, ^4^, ^5^, ^6^

**Figure 1:**
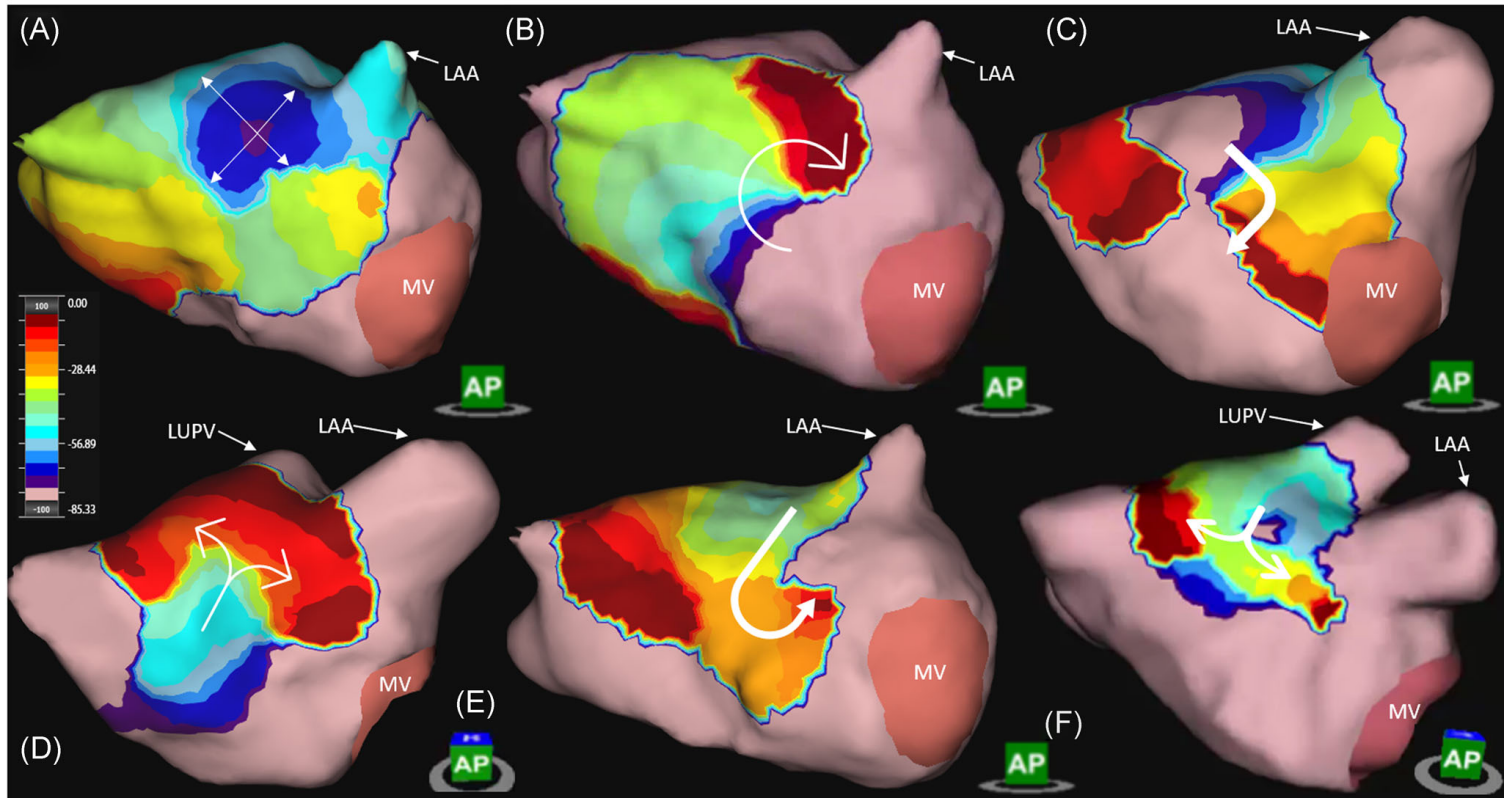
Dynamic AcQTrack analysis identifies each activation pattern including focal firing (FF) (A) characterized by radial activation from a central earliest point, localized rotational activation (LRA) (B) where smooth rotational activation of >270° is observed; and localized irregular activation (LIA) (C). LIA includes a range of specific patterns of activation, all characterized by changing wavefront direction of >90° (C–F, dynamic AcQTrack detection not shown). Color scale depicts the leading (red) to trailing edge (purple) of a wavefront with the full color spectrum occupying 80 ms. LAA, left atrial appendage; LUPV, left upper pulmonary vein; MV, mitral valve

Noncontact charge‐density mapping allows visualization of whole chamber activation. Ultrasound is used to generate a high resolution 3‐dimensional reconstruction of the atrial chamber anatomy based on a triangular mesh structure comprising constituent corners (termed vertices) of triangular faces, which form the unique points for calculation of inverse derived charge density signals. Wavefront patterns are scrutinized in real time at every vertex of the chamber surface (approximately 3500) by in an inbuilt application (AcQTrack; Acutus Medical). Nonplanar, complex localized patterns of propagation are identified and characterized as localized rotational activation (LRA), localized irregular activation (LIA) and focal firing (FF) (see Figure 1).^7^ To be classed as LRA, a smooth depolarization wavefront has to rotate 360° around a central point. LIA is characterized by a difference in angle between conduction that enters and leaves a confined region exceeding a threshold of 90° (and not meeting the criteria for LRA above). In contrast, FF is defined as activation of a primary vertex that precedes adjoining neighbors and extends centrifugally from this primary vertex.

A catheter ablation approach aimed at targeting these zones has been evaluated in one prospective observational study.^6^ Within this study, mapping durations of approximately 5 s were used to identify ablation targets, but little work has been done exploring the spatial and temporal stability of these electrophysiological phenomena and the properties of these regions in sinus rhythm, knowledge of which is crucial in developing an optimal approach. We have performed the first study employing simultaneous mapping of AF activation in both the left and right atria using the AcQMap system and sought to investigate the spatial stability between two separate 30‐s recordings of left and right atrial AF propagation and the effects of increasing duration of AF recording length on the degree of variability in mechanisms observed. Properties of atrial regions with the most stable patterns were explored using long and short cycle length pacing and electroanatomic voltage mapping in sinus rhythm.

## 2 METHODS

### 2.1 Patient selection

Patients between the ages 18–80 undergoing first time catheter ablation for either paroxysmal (*n* = 5) or persistent (*n* = 17) AF were recruited following appropriate ethical approval (REC reference 18/SC/0409, clinicaltrials.gov NCT03812601). Exclusion criteria included prior cardiac surgery, congenital cardiac abnormalities and severe valvular heart disease.

### 2.2 Electrophysiological mapping and ablation

**Figure 2:**
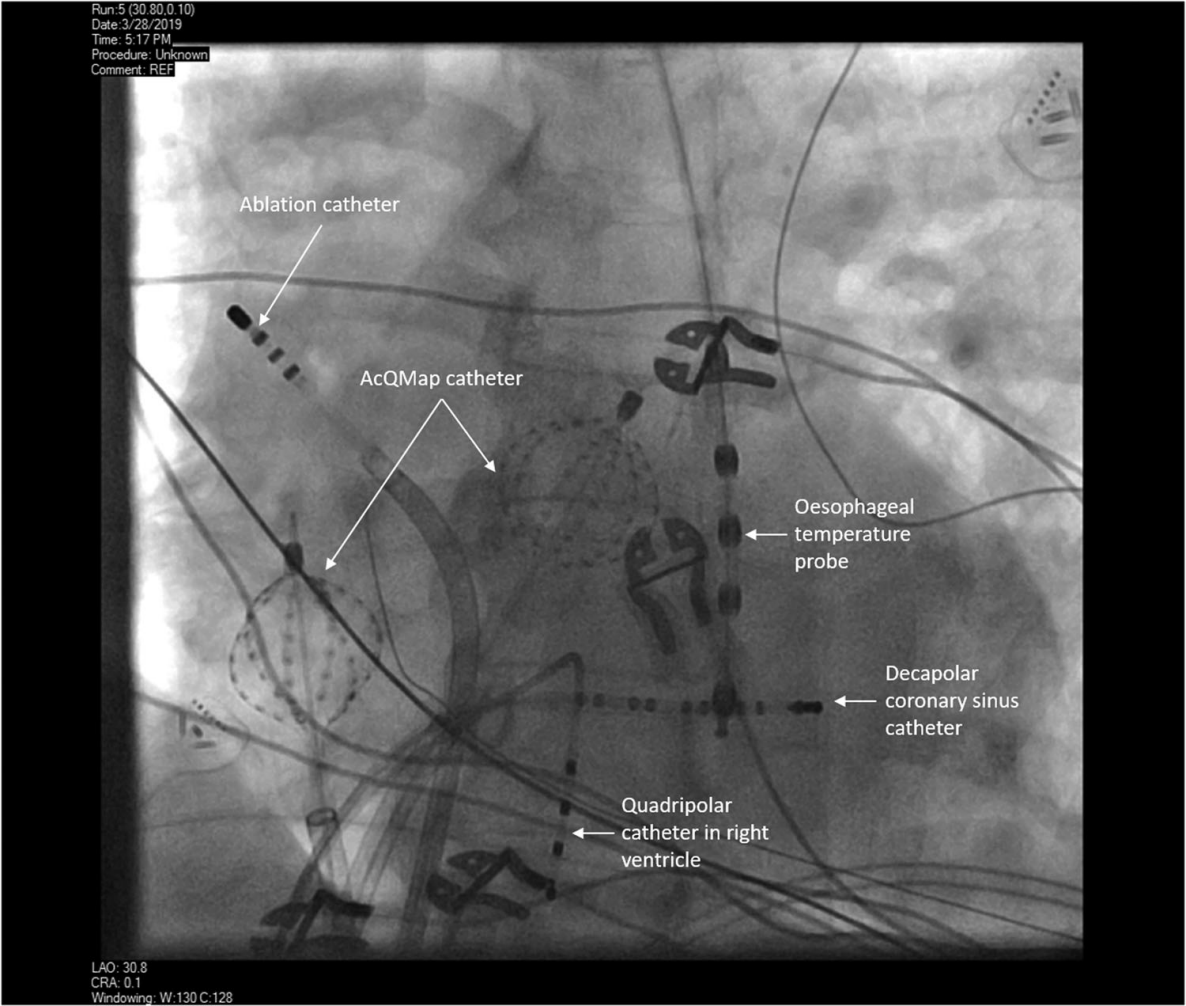
Fluoroscopic image of catheters positioned for simultaneous bi‐atrial noncontact mapping

Procedures were carried out under general anesthetic. With the exception of amiodarone, antiarrhythmic drugs were stopped a minimum of 5 days before the procedure. Venous access was obtained via bilateral femoral vein puncture under direct ultrasound guidance. Heparin boluses were administered before trans‐septal puncture followed by continuous heparin infusion to maintain an ACT >350 s. A decapolar catheter (Inquiry; Abbott Medical) was inserted into the coronary sinus through an AcQRef (Acutus Medical) sheath which includes a distal electrode used as a unipolar reference. The first AcQMap catheter was advanced over a 0.032 guide wire into the RA via an AcQGuide (Acutus Medical) sheath and ultrasound used to reconstruct the right atrial chamber anatomy as previously described.^7^ The ablation catheter (Tacticath; Abbott Medical) was advanced via an Agilis sheath into the left atrium across the single transseptal puncture site. The second AcQGuide sheath was then exchanged for the transseptal access sheath over the guide wire and a second AcQMap catheter advanced into the LA (see Figure 2). The LA anatomy was then generated with ultrasound. A circular mapping catheter (Inquiry Optima or Advisor Variable Loop; Abbott Medical) was used to undertake electroanatomic voltage mapping and guide pulmonary vein isolation.

In a subset of nine patients, activation maps were obtained using Supermap during pacing from up to three atrial sites (left atrial appendage, high right atrium, and proximal coronary sinus) with direct cardioversion used to restore sinus rhythm where necessary. Pacing consisted of a 4‐beat drive train at 800 ms cycle length followed by a single extrastimulus with coupling interval 20 ms above the effective refractory period and was mapped using the AcQMap Supermap algorithm. Additional details are provided in the supplementary methods.

In patients attending the procedure in sinus rhythm AF was induced using burst atrial pacing, otherwise all AF recordings were obtained before DCCV. Once AF was established, recordings of 2 min duration were generated and time alignment between systems facilitated using a below threshold pacing stimulus from the coronary sinus catheter of 4 beats at 1000, 800, and 600 ms intervals and 12 s rest period. Pulmonary vein isolation was performed using contact force guided radiofrequency ablation using 40–50 W (Tacticath ablation catheter; Abbott Medical). Simultaneous biatrial AF mapping was repeated following completion of PVI. Additional ablation and AF mapping was undertaken at the discretion of the operator.

### 2.3 Propagation map calculation and data export

Raw AcQMap electrode biopotential signals were visually inspected to identify outlying or corrupted signals, which were then manually excluded. A low pass filter at 100 Hz as well as a 50 Hz notch filter and smoothing algorithm were applied followed by selection of a QRS‐T wave template for subtraction. The AcQMap system allows operators to define separate mapping segments of any duration up to a maximum of approximately 14–15 s, limited by software processing capability. For this study, three consecutive 10‐s mapping segments were created for simultaneous left and right atrial mapping, synchronized using the coronary sinus low amplitude pacing spike. This process was repeated to create two 30‐s AF maps following splicing together of each 10 s segment. Propagation maps were calculated using the default timing method (based on ‐dv/dt of dipole signals), window width (for isochronal color bars) of 80 ms and time threshold of 70 ms (representing presumed minimum refractoriness). AcQTrack propagation patterns were calculated for each segment and data exported for offline analysis.

### 2.4 Propagation pattern quantification

Complex propagation patterns described above (LIA, LRA, and FF) were identified using the AcQTrack (Acutus Medical) system during AF to ensure objective classification of activation patterns, as outlined in the supplementary methods. Representative examples of these patterns are seen in Figure 1 and Videos [Link], [Link], [Link]. Every wavefront is scrutinized at each vertex of the anatomy. Planar wavefronts are discarded, whilst if the parameters for LIA, LRA, and FF are met within a discreet zone (300 mm^2^ for LRA and 200 mm^2^ for LIA) the vertices within this zone are highlighted and recorded by the system.

**Figure 3:**
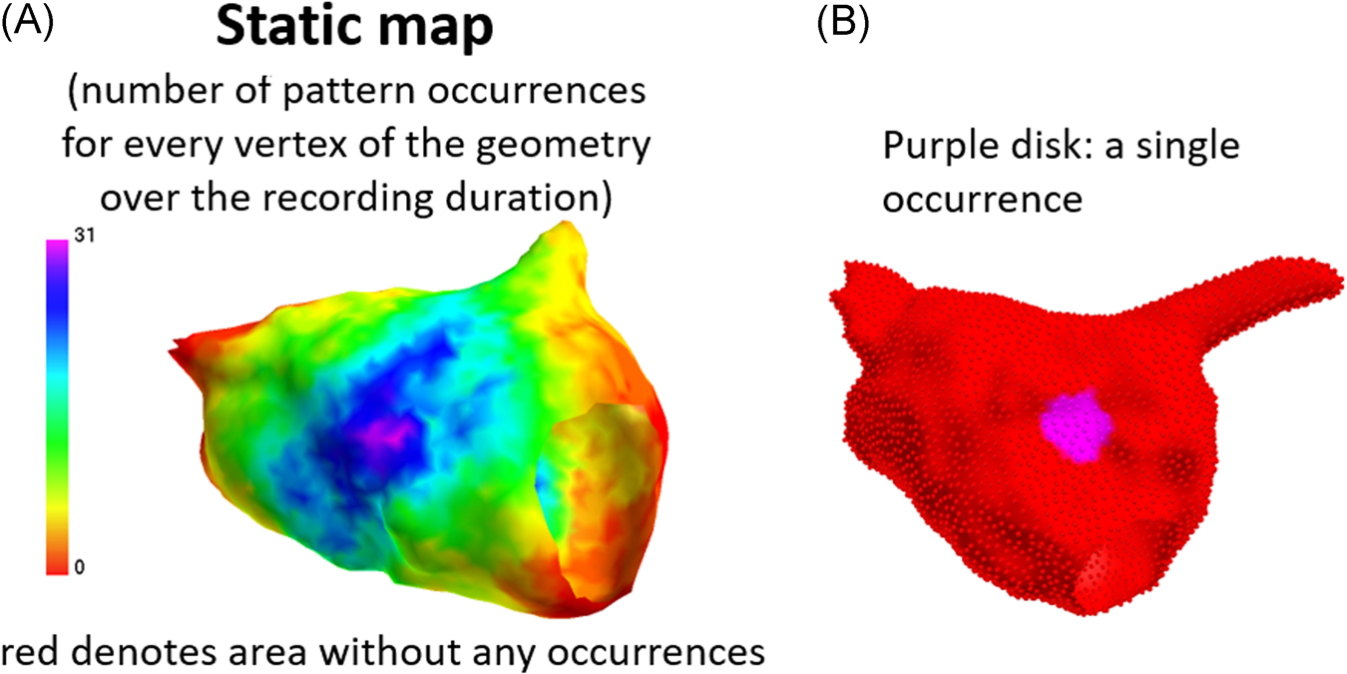
Method for AcQTrack pattern quantification. A static map is generated (A) quantifying all pattern occurrences at every vertex of the chamber anatomy. Each occurrence is identified in space and time as a single disc allowing calculation of the total number of occurrences, the percentage time they are present, and the proportion of the chamber surface area affected. See also Figure S3

These data were exported and analysed using a custom designed program to allow quantification. The process is outlined in Figure 3 and Figure S3. Initially, all AcQTrack data is exported to create a static map quantifying every pattern occurrence at each vertex of the chamber anatomy (approximately 3500 per chamber) for the entire recording duration (Figure 3A). Each single occurrence is signified by a disc that occurs in a specific region for the duration that pattern is present (Figure 3B). The number of these unique “discs” equates to the number of occurrences of the specified propagation pattern. When taken over the duration of the recording, the proportion of time in which these discs are detected on the chamber surface represents the time parameter. Similarly, the proportion of the chamber in which an occurrence is detected represents the surface area affected.

**Figure 4:**
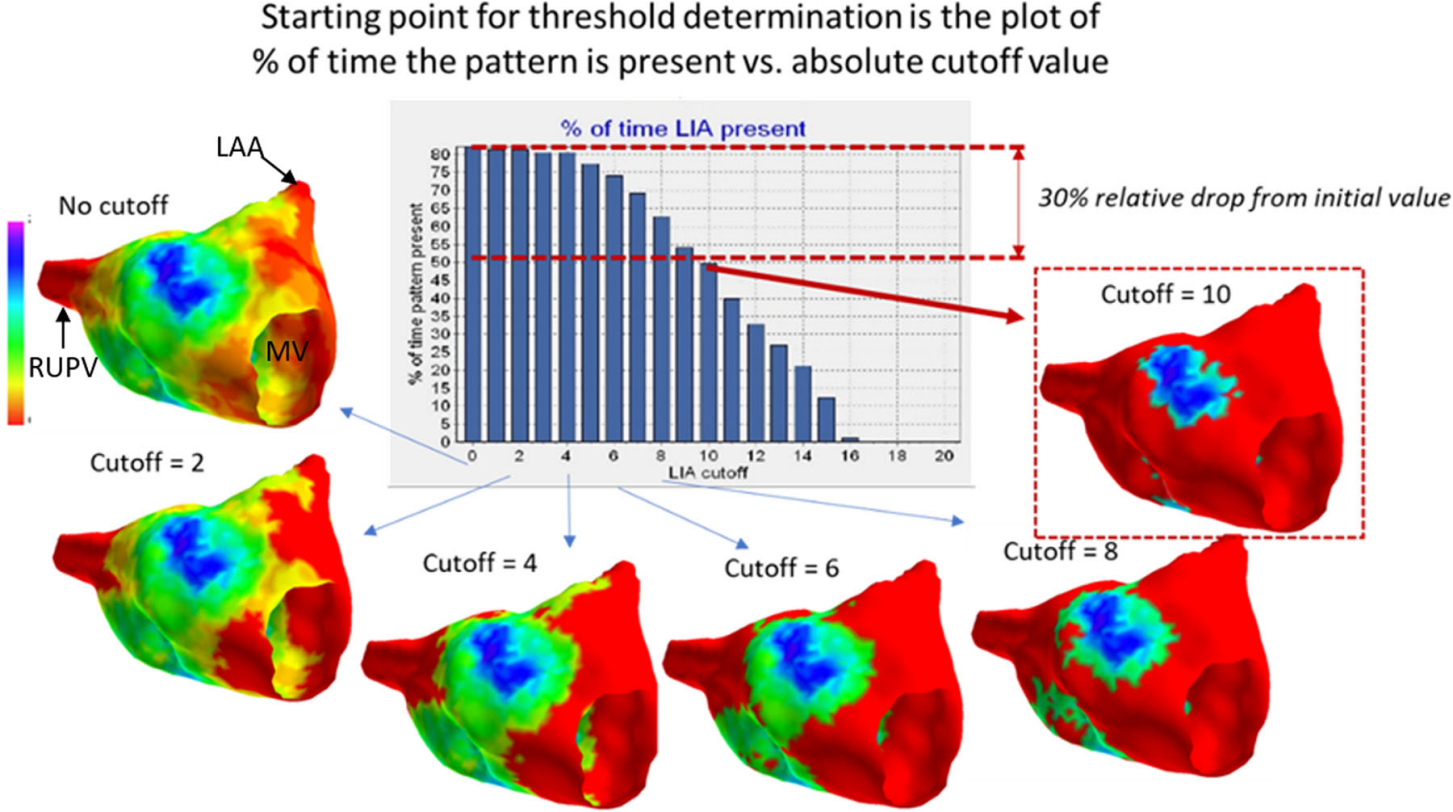
Threshold method. Cut off values for LIA are generated according to the total time the pattern is detected using thresholds gradually excluding more infrequent occurrences (see text for detailed explanation). LAA, left atrial appendage; MV, mitral valve; RUPV, right upper pulmonary vein

A cut off threshold is then applied to exclude outlying data and identify the localized region with the most repetitive pattern occurrences. The number of occurrences in each region and the proportion of time that they are present are known. These factors are used to determine the optimum threshold as outlined in Figure 4. The initial static map displays all occurrences with no cut off applied (zero on the X axis i.e., every occurrence is counted). The percentage of the recording time with the relevant pattern is shown on the Y axis. As the cut off is increased along the X axis (i.e., only regions with increasing numbers of occurrences are included), the proportion of time these are present decreases. A threshold is applied relative to the total time pattern occurrences are present. In the example in Figure 4, (for LIA) with no cut off, LIA was present for 85% of the mapping duration. Increases in the threshold resulted in a reduction in the percentage of time that LIA was present. A 10% threshold excludes LIA to the point at which only regions with LIA present for 75% of the recording duration are counted, which corresponds to more than six occurrences over the duration of the recording. Similarly, a threshold that corresponds to a 30% relative reduction from the initial maximum duration identifies the region with only the highest number of occurrences. Where discs representing a detection are overlapping at any time point (potentially representing a meandering central pivot or rotation point) these are counted as a single pattern detection. Once a cut off is applied, only occurrences with the geometric center of the “disc” within the specified zone are included for quantification and any occurrence detected within 5 ms of a preceding occurrence in the same location is excluded to avoid double counting.

### 2.5 Spatial stability

Spatial stability was assessed by correlating the frequency of propagation patterns (LIA, LRA, and FF) at each vertex of the chamber anatomy over two separate 30‐s recordings taken at baseline before any ablation. The value for the number of pattern occurrences were plotted for each vertex of the anatomy for each recording. A linear best fit line was plotted, and *R* ^2^ calculated to assess the strength of the linear correlation between the two mapping segments. Stability of regions with the most repetitive patterns identified using the 30% threshold value for each 30‐s map were compared using Cohen's kappa statistic.

### 2.6 Temporal stability/optimal mapping duration

We sought to identify the minimum mapping duration required to identify the sites that are shown over the full 30 s analysis to represent regions with the highest frequency and therefore most repetitive activation for each of the specific patterns described. The region with the highest frequency of each propagation pattern may be considered as reflecting an optimum target for ablation. This was identified using the 30% cut off to identify the relevant zone on maps of increasing duration up to 30 s. Each vertex of the anatomy contained within and outside this region (at 30% cut off) was used to calculate the kappa statistic to quantify the consistency between these zones against the zone identified during a full 30 s segment. Kappa values were calculated and plotted at both 1‐s and 5‐s increments with a value of 0.8 considered excellent consistency compared to the result obtained at 30 s. The duration at which a kappa value of 0.8 was reached was extracted for each map and used for comparisons.

In a subset of 15 patients, additional analyses were undertaken using alternative methods to confirm that the results obtained were consistent between methods used. LIA, LRA, and FF were quantified for occurrence frequency, percentage time present and percentage of the chamber surface area affected (for FF only frequency was assessed) at increasing durations, also in 1‐s increments up to 30 s. At each incremental recording duration, the percentage change in each variable was calculated. For occurrence frequency the results for every possible combination of maps of increasing duration within the 30‐s recording were compared (e.g., the frequency of a pattern was measured over 5 s and compared with all possible maps of 5‐s duration within the full 30‐s recording). For occurrence time and surface area a 5 s moving average at 1‐s increments was calculated. Heatmaps were created for each pattern allowing a visual representation of the effect of duration on variability.

### 2.7 Electroanatomic voltage mapping and conduction heterogeneity

Left and right atrial geometries created using Ensite Precision were exported and fused with the AcQMap anatomies using fiducial points and a nearest neighbor approach to assign bipolar voltage amplitude to each site on the AcQMap chamber enabling comparison between sites according to identified activation patterns.

Activation time maps obtained using Supermap were exported and analysed offline. The local activation time difference (LAT) for a 5 mm radius from every vertex of the anatomy was calculated for each map and the largest value taken for each point to generate a histogram. The median LAT difference (MAT) and the conduction heterogeneity index (CHI) (95th percentile—5th percentile/median) was calculated within regions shown to demonstrate stable high frequency activation patterns during AF as described previously^8^ and the effect of extrastimulus pacing on the CHI calculated in these regions compared to the remainder of the chamber.

### 2.8 Statistics

Continuous variables are expressed as mean ± *SD* or median and interquartile range depending on distribution. Between group comparisons were performed using independent samples *t* test or Wilcoxon rank sum test depending on distribution as assessed using the Kolmogorov–Smirnov test. A two‐sided *p* value of <.05 was considered significant. Statistical analysis was performed using SPSS (IBM v25) or Matlab (R2019a, MathWorks) and figures created using Matlab.

## 3 RESULTS

### 3.1 Patient characteristics and map segments obtained

The characteristics of all patients recruited to the study are outlined in Table 1. One patient (Patient 9) was excluded from analysis as only organized atrial tachycardia could be induced when attending for the procedure. In all but one patient (Patient 12), two 30‐s maps of AF were obtained at baseline. Patient 12 had varying QRS morphology as a result of left bundle branch block and only one 30‐s segment could be obtained with sufficient quality of QRS‐T wave subtraction to allow analysis. Maps following pulmonary vein isolation were obtained in 12 patients and a further 8 recordings were obtained following nonpulmonary vein ablation providing a total of 62 simultaneous AF recordings in the left and right atrium. Mapping duration and temporal variability analysis was performed on all maps obtained (a total of 124 maps including both chambers). Spatial variability analysis was performed by comparing the two 30‐s maps obtained at baseline in the 20 patients.

**Table 1:** Patient characteristics

Characteristic	Distribution
AF type	16 Persistent, 5 paroxysmal
Sex	41% Female
Age	61 (56–66)
BMI (kg/m^2^)	30 (26–33)
CHADs‐VASc	1.3 ± 1.3
Prior antiarrhythmic drugs	Sotalol: 2 (9.5%)
	Amiodarone: 7 (33%)
	Flecainide: 4 (19%)
LA diameter (mm)	43 (38–51)
LV ejection fraction (%)	60 (55–60)
Time since AF diagnosis (months)	48 (20–60)
Rhythm at baseline	SR: 11, AF: 10

*Note:* Data are expressed as n (%), medians, 1st and 3rd quartiles or mean ± *SD*. Patient 9 was excluded from analysis as only atrial tachycardia could be induced.

Abbreviations: AF, atrial fibrillation; BMI, body mass index; LA, left atrium; LV, left ventricle; SR, sinus rhythm.

### 3.2 Spatial variability

**Figure 5:**
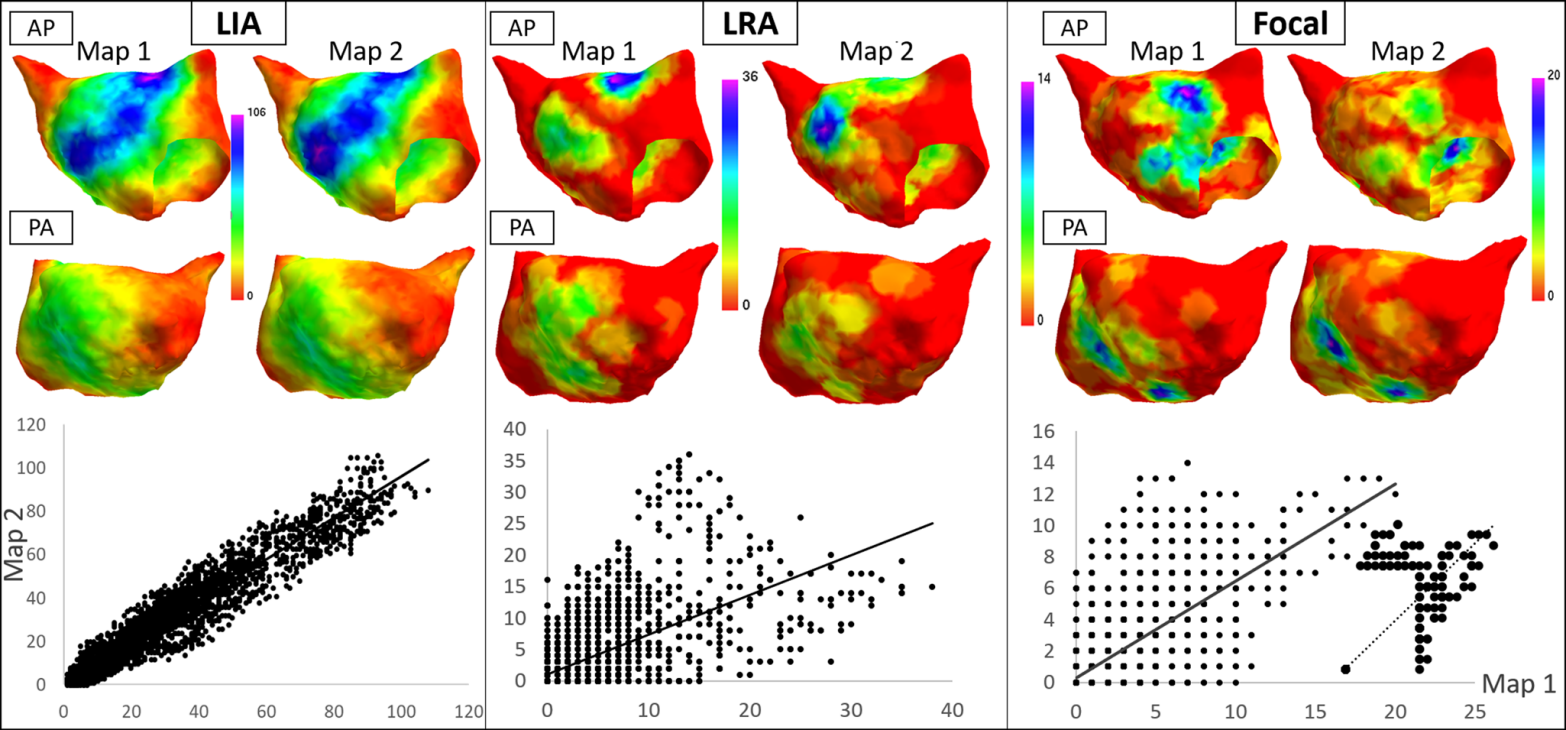
Antero‐posterior (top row) and postero‐anterior (lower row) views the left atrium showing the density and distribution of localized irregular activation, localized rotational activation and focal firing (red = no occurrences, purple = highest density) in Patient 10. Each pair of images represent 2 sequential 30‐s maps. Graphs below represent correlation plots for each anatomy vertex (1st map X axis, 2nd Y axis) with *R* ^2^ for LIA 0.92, LRA 0.39, and FF 0.49. Inset graph shows correlation for regions with FF frequency ≥1 every 3 s on the first map with *R* ^2^ of 0.74. FF, focal firing; LIA, localized irregular activation; LRA, localized rotational activation

LIA demonstrated the greatest spatial stability between each 30‐s recording with *R* ^2^ of 0.83 (0.71–0.88) across all maps analysed. This was consistent across both chambers with values of 0.81 (0.75–0.88) and 0.84 (0.61–0.88) in the LA and RA, respectively. Median *R* ^2^ for LRA and FF were 0.39 (0.24–0.57) and 0.64 (0.54–0.73), respectively. Multiple low frequency FFs were seen widely distributed across the atrial surface (see Figure 5). For high frequency FF, defined as occurring ≥10 times over the 30 s, *R* ^2^ was 0.83 (0.68–0.85). Detailed results are outlined in Table 2.

**Table 2:** *R* ^2^ values revealing the spatial stability of propagation patterns across two repeated mapping segments of 30‐s duration

	LIA		LRA		FF		High frequency FF	
	LA	RA	LA	RA	LA	RA	LA	RA
*R* ^2^ value	0.81 (0.75−0.88)	0.84 (0.61–0.88)	0.39 (0.25–0.53)	0.41 (0.23–0.58)	0.58 (0.50–0.76)	0.66 (0.60–0.73)	0.80 (0.66–0.87)	0.83 (0.76–0.85)

*Note:* Data are expressed as median (interquartile range).

Abbreviations: FF, focal firing; LIA, localized irregular activation; LRA, localized rotational activation.

Stability of regions across both maps with the highest frequency patterns identified at 30% cut off was also greatest for LIA in both the LA and RA. Cohen kappa statistic for LIA in the LA and RA, respectively, was 0.75 (0.64–0.78) and 0.75 (0.58–0.79). Full kappa statistic results for all patients are outlined in Table 3.

**Table 3:** Cohen kappa statistic comparing the consistency of regions identified with the highest frequency of LIA, LRA, and FF at the 30% cut off in both the LA and RA

	LIA		LRA		FF	
	LA	RA	LA	RA	LA	RA
Median (interquartile range)	0.75 (0.64–0.78)	0.75 (0.58–0.79)	0.43 (0.33–0.49)	0.40 (0.28–0.47)	0.56 (0.47–0.64)	0.60 (0.53–0.63)

*Note:* Data are expressed as median (interquartile range).

Abbreviations: FF, focal firing; LIA, localized irregular activation; LRA, localized rotational activation.

The anatomical regions with maximal LIA were the anterior and posterior LA (in 46% and 27% of maps, respectively) and the lateral and septal RA (in 46% and 32%, respectively). LRA showed similar distribution with the zone of highest LRA frequency in the posterior LA in 41% and the anterior LA in 34%. In the RA, the lateral wall was the most common site (in 37%) followed by the septum and posterior walls (each in 24%). A similar distribution was observed for FF, most commonly involving the anterior and posterior LA (in 34% and 29%, respectively), followed by the LA septum (20%). In the RA, the highest frequency of FF was seen in the septum in 59% and the lateral wall in 22%.

### 3.3 Mapping duration results

**Figure 6:**
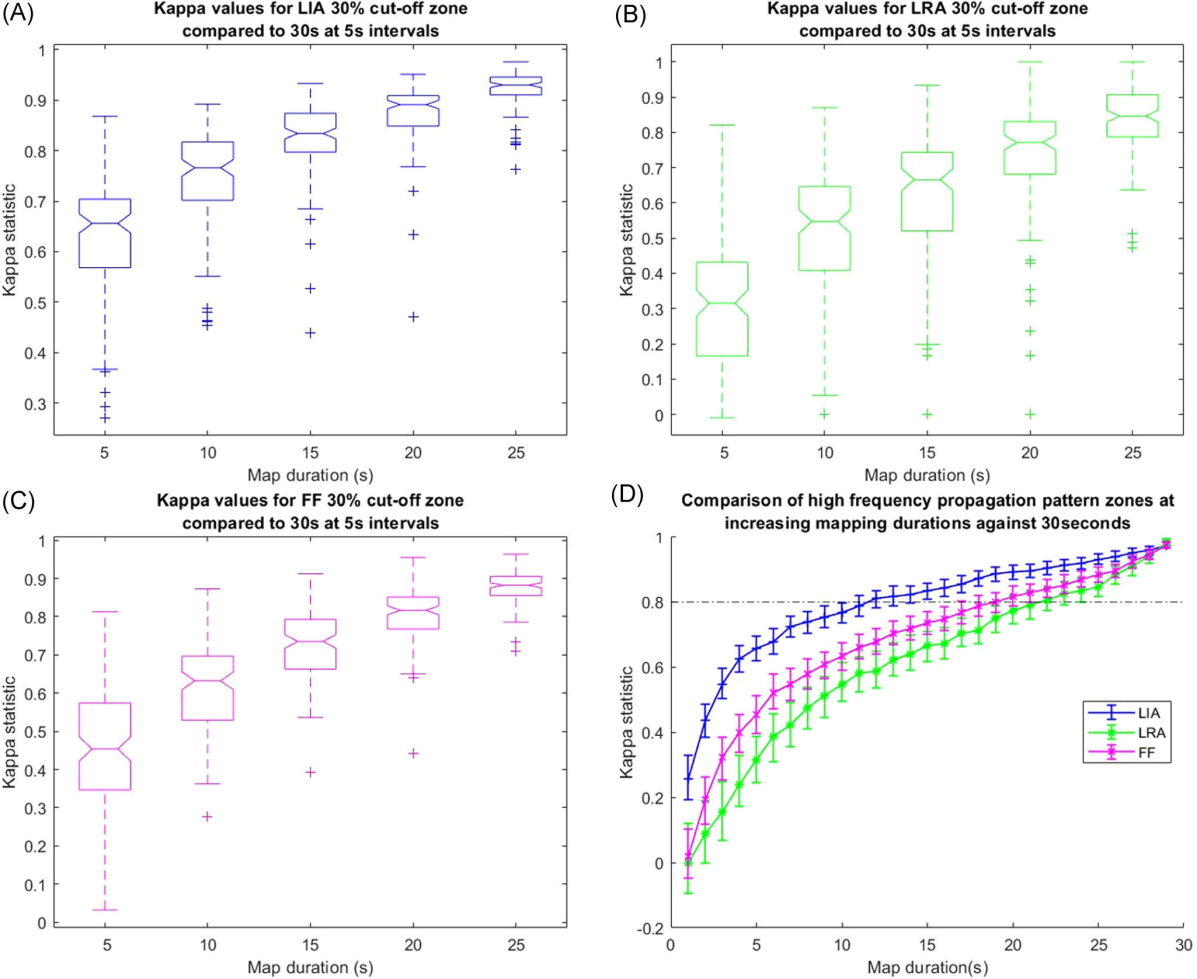
Boxplots (A–C) showing the distribution of kappa values in 5‐s increments for regions with high frequency (at the 30% cut off) LIA, LRA, and FF respectively compared to the result after 30 s. Kappa values for the LIA, LRA, and FF are shown at 1‐s increments in (D) showing the point at which the kappa value (representing a very good level of agreement) reaches 0.8. FF, focal firing; LIA, localized irregular activation; LRA, localized rotational activation

Figure 6 demonstrates the effect of increasing map duration on kappa statistic values for LIA, LRA, and FF respectively compared to maps of 30 s. Analysis was conducted on all 124 recordings (62 in the LA and 62 in the RA) obtained. LIA showed the highest kappa value at all time periods with a value of 0.66 (0.57–0.70) at 5 s, rising to 0.93 (0.91–0.95) at 25 s. LRA demonstrated the lowest value of 0.32 (0.17–0.43) at 5 s, with a maximum of 0.85 (0.79–0.91) at 25 s. A kappa value of 0.8 can be considered excellent correlation. Figure 6D shows kappa values at 1‐s increments for LIA, LRA, and FF. A value for 0.8 was reached by 12 s (interquartile range [IQR]: 6) for LIA compared to 19 s (IQR: 7) for FF (*p* < .0005) and 22 s (IQR: 8) for LRA (*p* < .0005).

The duration of mapping required to achieve a kappa value of 0.8 was compared between the left and right atria, in patients with paroxysmal and persistent AF, before and after pulmonary vein isolation and in patients on or off amiodarone. There was no difference for any parameter between the left and right atria. In patients with persistent AF, LIA stabilized earlier than in patients with paroxysmal AF (11 ± 6 vs. 15 ± 4, difference 4 s, 95% confidence interval [CI]: 0.6–5.6, *p* = .004). There was no difference between any groups for stability of LRA. The frequency of FF stabilized after 17.2 ± 5.6 s following pulmonary vein isolation compared to 19.1 ± 3.7 s before pulmonary vein isolation, a difference of 1.9 s (95% CI: 0.3–3.6, *p* = .023). Full results for all comparisons are in supplementary figures S6 and S7 and Tables S1–S3.

Heatmaps for the subset of 15 patients are shown in supplementary figure S8. When measured as either a frequency, proportion of time the propagation pattern was present or a proportion of the atrial surface area that patterns occurred, variability in LIA fell rapidly, followed by FF and LRA. A significant degree of variability in LRA patterns was observed, particularly when measured as a proportion of the atrial surface affected up to durations of 20–25 s.

### 3.4 Voltage and conduction properties

The median distance between merged chamber surfaces was 6.0 mm (IQR: 3.4–10.7). The voltage in regions of high frequency LIA was 1.09 mv (IQR: 0.55–1.94) compared to 1.07 mv (IQR: 0.51–1.94) in the remainder of the chamber (*p* = .9936).

A total of 53 paired maps at long and short cycle length were obtained for the LA and RA in a subset of 9 patients (in 1 participant only 2 sites were obtained due to AF induction). Having identified LIA as spatially stable, conduction properties during pacing were analysed within these regions. The MAT across all maps obtained in regions with high frequency LIA was 7.5 ms (IQR: 6.6–8.9) compared to 6.0 ms (IQR: 5.2–7.7) in the remainder of the chamber, a statistically significant difference of 1.5 ms, *p* < .0005. Extrastimulus pacing resulted in a significant increase in CHI in regions of high frequency LIA from 3.3 (IQR: 2.3–4.4) to 4.0 (IQR: 3.1–5.4) (*p* = .0480), but no increase in the remainder of the chamber (*p* = .4636) as shown in Figure S9.

## 4 DISCUSSION

This study demonstrates that regions with LIA patterns show high spatiotemporal stability. In contrast rotational activation patterns, closest to the “rotors” identified using other mapping techniques, show the least spatiotemporal stability. Regions of high frequency FF are relatively more stable whereas infrequent FF is not. Mapping durations of 20–25 s are required to identify all temporally variable propagation patterns although shorter durations will identify the most stable LIA and FF. Although bipolar voltage amplitude in these regions is normal, they demonstrate an increase in conduction heterogeneity during short coupled extrastimulus pacing.

The aim of technologies designed to facilitate electrophysiological mapping and ablation of AF mechanisms is to identify repetitive patterns within a characteristically disorganized rhythm. The total duration analysed has a significant impact on how a repetitive pattern is defined and there have been limited efforts previously to determine the optimum duration required. Shi et al.^9^ have previously described the distribution and frequency of charge density activation patterns within the LA but used short R‐R interval segments within a 30‐s recording precluding a robust assessment of either spatial or temporal stability. Studies often do not report the duration of AF mapped but may report that patterns identified are stable over several minutes and separate recordings.^1^, ^10^, ^11^ Other studies have used recording durations of between 10 s and 5 min,^3^, ^5^, ^12^ whilst an analysis of the mapping duration required to identify sites at which ablation terminated AF suggested a duration of between 4 and 30 s was required.^13^ However, the shorter duration suggested relied on a driver definition requiring only very transient occurrence (present for >0.4 s within a 4 s period) insufficient to result in discrimination between other regions of similarly low frequency when analysed prospectively, without the prior knowledge of the effect of ablation at that site. Of note a retrospective analysis where 5 min initial recordings were used found that 89% of the mechanistic sites identified were also seen when 30‐s recording durations were analysed.^14^ However, shorter durations than this were not assessed. It may be revealing that when only 10‐s recording durations have been chosen, in the study by Child et al.^3^ using a technique of basket contact mapping and phase singularity analysis, spatially stable patterns were not identified. Rotational activation patterns demonstrate the least stability between and during recordings, with 10 s of mapping showing only very moderate correlation with the results obtained from 30‐s mapping (kappa 0.55) and a variability in rotational activation pattern frequency of approximately 20% at a duration of 10 s. The fact that the region that appears to represent the highest frequency of LRA after 10 s of analysis is significantly different from the region identified when longer durations are used highlights the transient and variable nature of these activation patterns. Of note, of course, is that durations beyond 30 s were not assessed and it may be that accuracy improves yet further if longer analyses are performed.

Traditional electrophysiological assessment has involved mapping of either the endocardial or epicardial surfaces. There is increasing recognition that the remodeling involved in the development and progression of AF is a three‐dimensional process resulting in activation time differences between atrial surfaces.^15^, ^16^ In this context, epicardial propagation that results in local breakthrough conduction will manifest as a focal activation pattern on the endocardial surface. The sites of epicardial breakthrough are likely to either be randomly distributed, if arising from chaotic 3‐dimensional propagation, or recur at specific sites where the remodeling process promotes breakthrough to the endocardial surface. Sporadic focal activations and random breakthroughs are likely to display minimal consistency across recordings whilst high frequency activations or sites of recurrent breakthrough are likely to be consistent. This was supported by the finding of much greater correlation at high frequency sites (*R* ^2^ value 0.83, IQR: 0.17) than when all activations are considered (*R* ^2^ 0.64, IQR: 0.19). However, distinguishing between a site of recurrent breakthrough and true focal activation is not possible using the mapping methods described here. There similarly appears to be earlier stabilization of FF variability following pulmonary vein isolation. This suggests a greater degree of stability in nonpulmonary vein sites of focal activation.

The spatial consistency of LIA detection between separate recordings is illustrated in Figure 5. Bipolar voltage amplitude in these regions is normal, which suggests that the activation properties observed are not the result of dense fibrosis. However, bipolar voltage amplitude is a relatively crude tool and is highly dependent on both rate and vector of activation.^17^ Studies using late gadolinium enhanced magnetic resonance imaging reveal patchy areas of fibrosis out of keeping with the burden seen on voltage mapping studies^18^, ^19^ suggesting the existence of interstitial fibrosis that is not revealed by measuring bipolar voltage amplitude. The MAT during pacing within LIA zones was longer, suggestive of slower conduction velocity, and short coupled extrastimulus pacing resulted in an increase in CHI in these regions that was not observed in the remainder of the chamber. Although these sites may represent anatomically normal regions of changing fiber orientation resulting in anisotropic conduction, they may represent disrupted conduction caused by underlying atrial interstitial fibrosis resulting in fiber disarray and rate dependent conduction abnormalities that manifest as local irregular activation patterns during AF. In a study by Walters et al.^20^ using surgically placed epicardial plaques in patients with longstanding persistent AF, disorganized activation was frequently observed, which did not satisfy criteria for either rotors or focal activations but was stable over multiple recordings of 10 s duration taken over a period of 10 min. This disorganized activation may represent similar propagation patterns to the irregular activation observed using charge density mapping, which was similarly stable even at short mapping durations. Walters also reported that rotors were frequently transient, in keeping with the results outlined here.

Both the nonhierarchical multiple‐wavelet hypothesis and the competing “mother‐rotor,” or focal driver, hypothesis describe a process of wave‐break in the formation of fibrillatory wavefronts involved in maintenance of cardiac fibrillation.^21^, ^22^ Tissue homogeneity is thought to play a significant role in the susceptibility to fibrillation^23^ with regions of structural inhomogeneity likely responsible for the wave‐break that results in fibrillatory conduction.^24^ The anatomical regions demonstrating stable LIA patterns identified in this study may therefore reflect sites of structural heterogeneity responsible for wave‐break, and therefore play an important role in AF maintenance.

Importantly, this study was not designed to assess ablation strategy or effectiveness and is not able to determine the impact of the phenomena identified on AF maintenance. This requires further detailed work. However, an understanding of the transient properties of rotational activity and low frequency focal activations observed in short mapping segments is crucial to designing ablation strategies that can be tested in clinical trials and suggests they are unlikely to occur as a result of anatomical substrate, such as scar or myofibre architecture. Targeting a fixed therapy to transient, migratory activation patterns is likely to be ineffective. The effect of atrial size on differences in activation patterns detected was also not assessed and would be of limited value given a relatively small sample size. Although there is some variation in correlation between contact unipolar electrograms and inverse derived electrograms, the distance of the atrial endocardium from the recording electrodes of the AcQMap catheter significantly influences this and may therefore have some impact on the activation patterns detected.^25^ However, a significant reduction in correlation occurs at distances greater than 40 mm. The median LA diameter of patients in our study was 43 mm resulting in significantly lower radial distances from the AcQMap catheter and, therefore, greater accuracy in reconstructed inverse electrograms.

## 5 CONCLUSION

Charge density mapping facilitates identification of complex patterns of wavefront propagation during atrial fibrillation. Although irregular activation patterns characterized by changing wavefront direction, and high frequency FF are spatially stable, rotational activations are transient and meandering, with low spatial stability. The duration of mapping recording used significantly impacts the results obtained. A minimum duration of 20 s is required to identify regions of repetitive but transient rotational activation whilst shorter segments will accurately reveal regions with high frequency irregular and focal activation. These stable regions of irregular activation may best reflect underlying atrial structural abnormalities and represent important sites for catheter ablation approaches.

## CONFLICT OF INTERESTS

Michael TB Pope has received honoraria and support for conference attendance from Acutus Medical. Pawel Kuklik provides consultancy services to Acutus Medical. Timothy R Betts has received honoraria from Acutus Medical and is a member of their Medical Advisory Board. The other authors declare that there are no conflict of interests.

## Supporting information

Supplementary information.

Supplementary information.

Supplementary information.

Supplementary information.

## Data Availability

Data are available from the corresponding author upon reasonable request.
