# Supplementary material for: Spatial and temporal variability of rotational, focal, and irregular activity: Practical implications for mapping of atrial fibrillation
Source: J Cardiovasc Electrophysiol. 2021 Jul 28;32(9):2393–403. doi: 10.1111/jce.15170 (PMC9290790; doi:10.1111/jce.15170)
Supplement: Supplementary file 4 — Supplementary information. [file JCE-32-2393-s002.docx]

**Supplementary methods:**

**Propagation pattern characterisation**

Propagation patterns described are identified using AcQTrack, an integrated platform within the AcQMap system. The propagation history map is first generated based on virtual dipole signals from each of approximately 3,500 vertices on the chamber surfaces. This map allows visualisation of wavefronts over the atrial surface. AcQTrack evaluates the propagation of these wavefronts to identify specific patterns of activation. Every vertex of the chamber is continuously analysed during the display of the propagation history map thereby allowing real-time identification of regions of interest, which can be displayed as both a dynamic map (where each activation pattern is highlighted during payback of the propagation history) and a cumulative map, where a sliding scale allows adjustment to the display according to the frequency of each pattern detected at any localised site. Patterns of activation identified include focal firing (FF), localised irregular activation (LIA) and localised rotational activation (LRA), with the specific algorithm used for their detection described below. Wavefronts that do not meet these definitions (for example smooth planar wavefronts) are discounted.

**Focal firing (FF)**

- The focal activation algorithm determines whether an activation at a vertex came from a previous cardiac wavefront, or whether activation spontaneously started from the current activation. Focal activation is detected at a vertex if an activation is earlier than its neighbors’ activation by at least 2-5 ms (default 3ms), and conduction spreads outward from the early activation.
- Activations are connected as a wavefront if the time difference between the two activation times would produce a conduction velocity greater than 0.05 m/s.

**Localised irregular activation (LIA)**

- The localised irregular activation algorithm computes the difference in angle between cardiac conduction entering and leaving a confined region, as illustrated in figure S1. If the angle difference of conduction entering and leaving a confined region exceeds 90 degrees, localised irregular activation is detected in the region.
- An area of approximately 200-300 mm^2^ is considered a confined region
- Wavefronts are considered to be passing through the region if the activation times differences between the border of the confined region and the central vertex would result in a conduction velocity between 0.3 m/s to 3.0 m/s.
- Activations are grouped into entering and leaving the region based on the activation time with comparison to the central vertex. A mean conduction vector entering the region and leaving the region are then computed. Angle difference between the vector entering and leaving the region are computed and if the difference exceeds 90 degrees, LIA is detected.


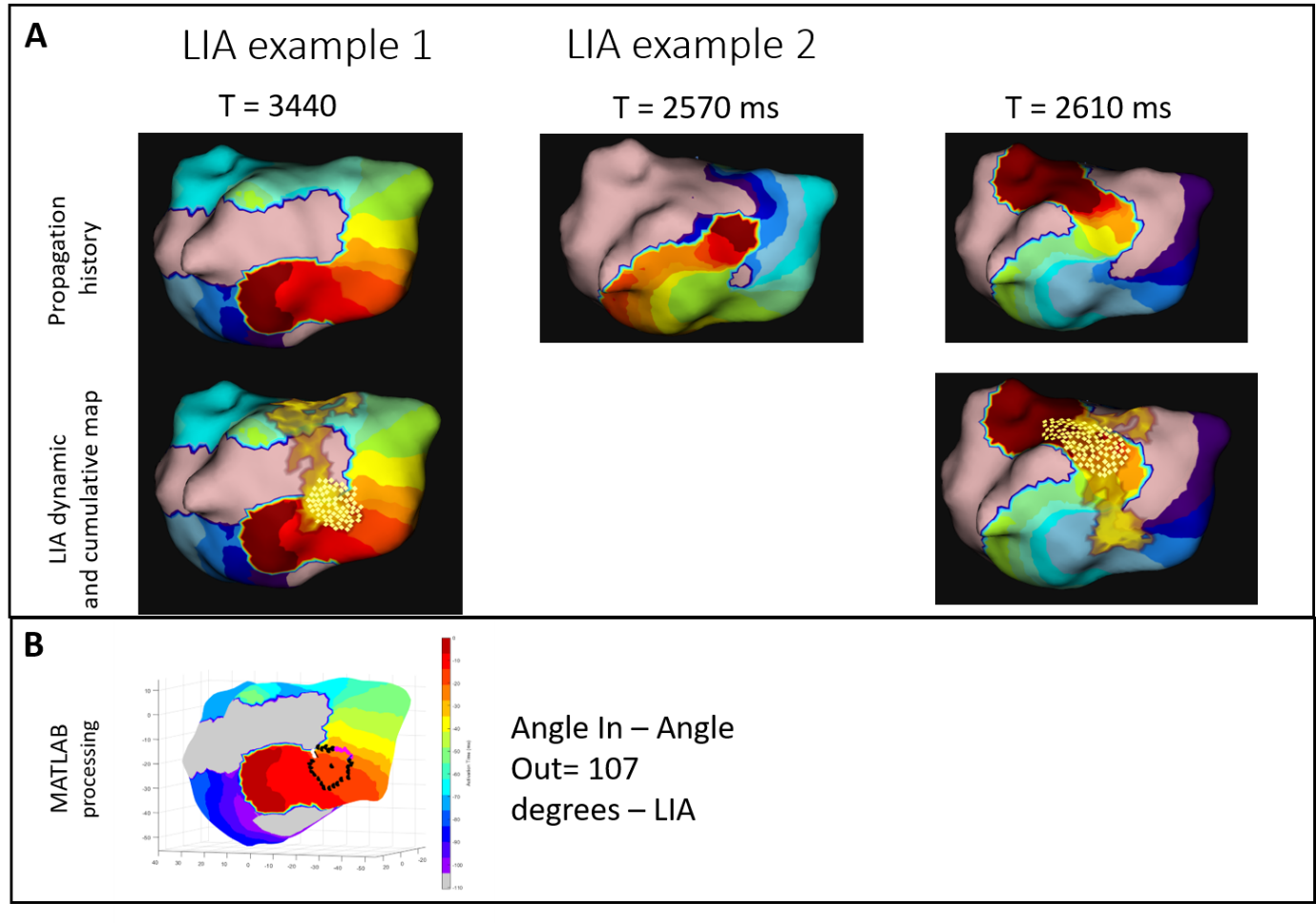
**Figure S1** Examples of patterns characterised as LIA are shown in A. Top row shows static images of a propagation history map taken at 3 time points with the row below including the dynamic view of LIA detection (yellow dots) and the cumulative map (yellow patch overlay) highlighting a region over the posterior wall where a frequency of LIA above a specified (user defined) threshold was detected. Panel B illustrates the computational processing that results in LIA classification for example 1. Red denotes the leading edge of the wavefront and purple the trailing edge. The time difference used for this display can be adjusted manually (here it is set to 100ms).

**Localised rotational activation (LRA)**

- The localised rotational activation algorithm computes the degrees of conduction propagation around a central point by summing the angle differences of sequential conduction velocity vector directions around the central point, as illustrated in figure S2. If the rotational angle of conduction vector changes exceeds 270 degrees, equating to total angle of propagation change of 360 degrees, rotation is detected at the central point. An area of approximately 200-300 mm^2^ around the central point is considered.
- To ensure smooth propagation around the central point, an r^2^ of a linear fit of activation time to position around the central vertex must exceed 0.7.
- Conduction velocity vector directions changes cannot exceed 45 degrees per position change around the vertex
- Activation time difference around the central obstacle must be greater than 50ms.


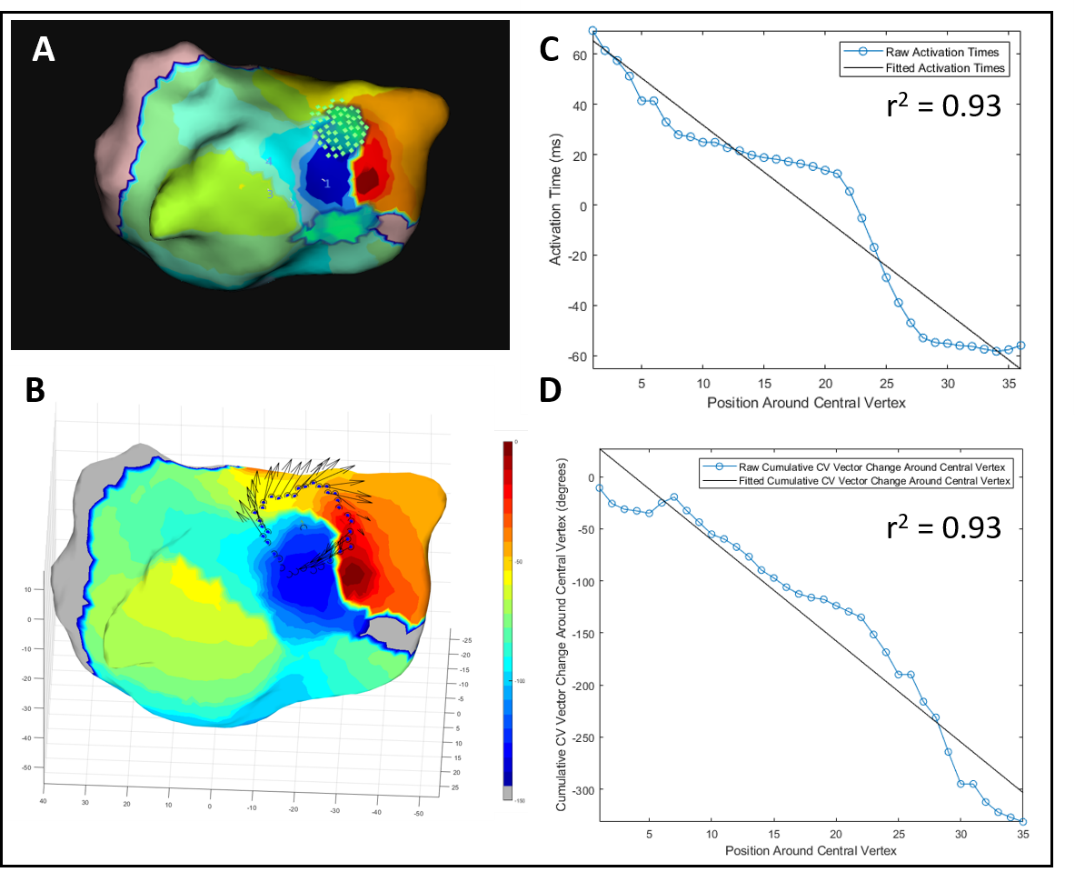


**Figure S2** (A) shows a static propagation history map including the dynamic view of LRA detection (green dots) and the cumulative map (green patch overlay) highlighting a region where a frequency of LRA above a specified (user defined) threshold was detected. (B) illustrates the processing and where activation times (C) and vectors (D) are plotted at points around a central vertex within the algorithm.

**Figure S3** Method for AcQTrack pattern quantification. A static map is generated (A) demonstrating all pattern occurrences. Each occurrence is identified in space and time (B-C) allowing calculation of the **
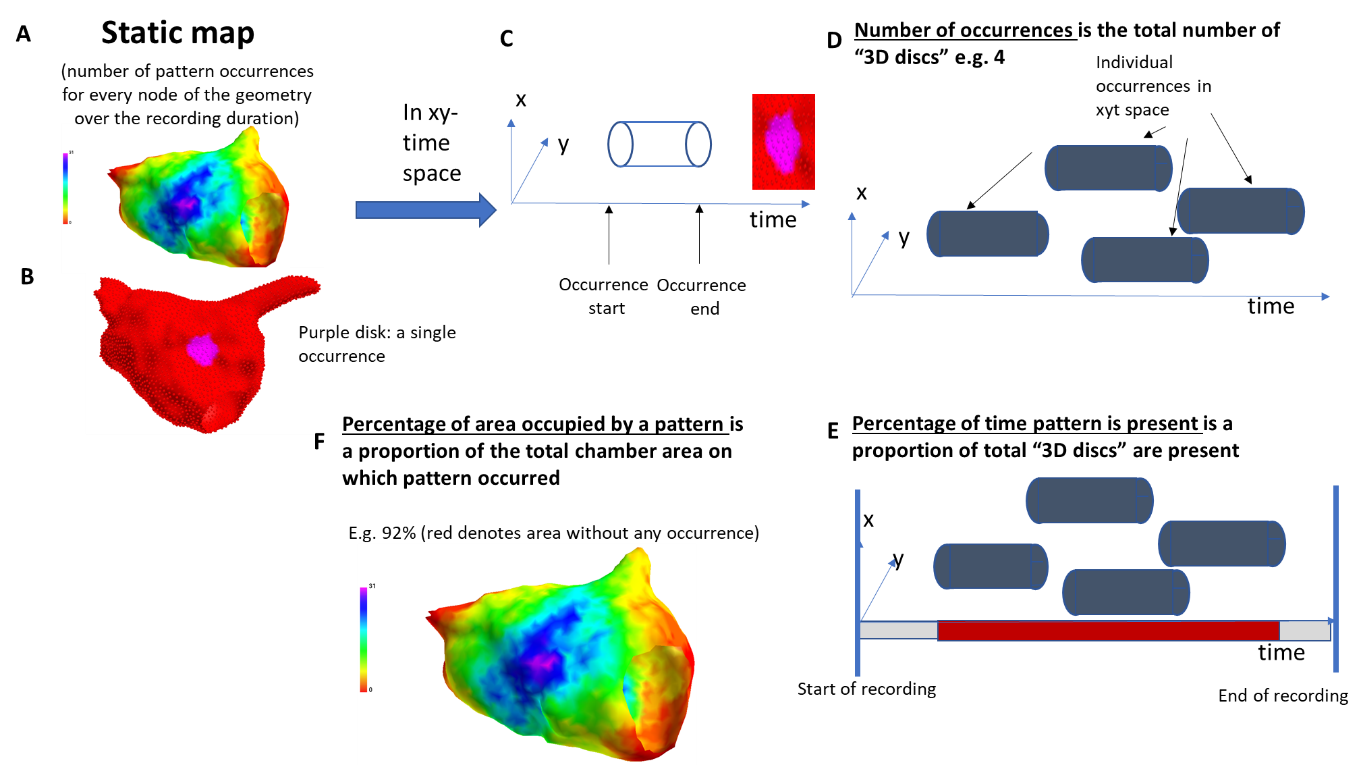
**total number of occurrences (D), the percentage time they are present (E), and the proportion of the chamber surface area affected (F).

**Electroanatomic Voltage Mapping and Geometry Registration**

Electroanatomic voltage mapping was performed in sinus rhythm with DCCV used following collection of all AF data in patients attending the procedure in AF. If DCCV was unsuccessful then data was obtained during AF. Mapping was performed using a circular mapping catheter (Inquiry Optima; 20x 1mm band electrodes with 2mm tip electrode and 1-4.5-1mm electrode spacing, or Advisor Variable Loop 20x 1mm electrodes with 1-4-1 spacing, Abbott Medical) and the Ensite Precision electrophysiological mapping system (Abbott Medical). Interpolation threshold was set to 7mm with internal and external projection limited to 5mm.

Bipolar voltage amplitude data together with chamber geometry was exported and re-constructed offline using a custom designed software application. Voltage points collected within the pulmonary veins and left atrial appendage were excluded as these regions are not visualised using the AcQMap system and provide non-representative results for comparison. Bipolar amplitude was measured from peak to peak in local electrograms. AcQMap and Precision anatomies were co-registered using a system of anatomical fiducial points and scaling of the Precision anatomy to minimise the average distance between chamber surfaces as shown in figure S4. Each vertex of the AcQMap anatomy was aligned with its nearest neighbour point on Precision to allow comparison and the corresponding voltage amplitude assigned to that vertex. Regions of high frequency LIA identified using the 30% cut off threshold were identified and the mean bipolar voltage amplitude corresponding to these regions was compared to the average for the rest of the chamber using the paired t-test.


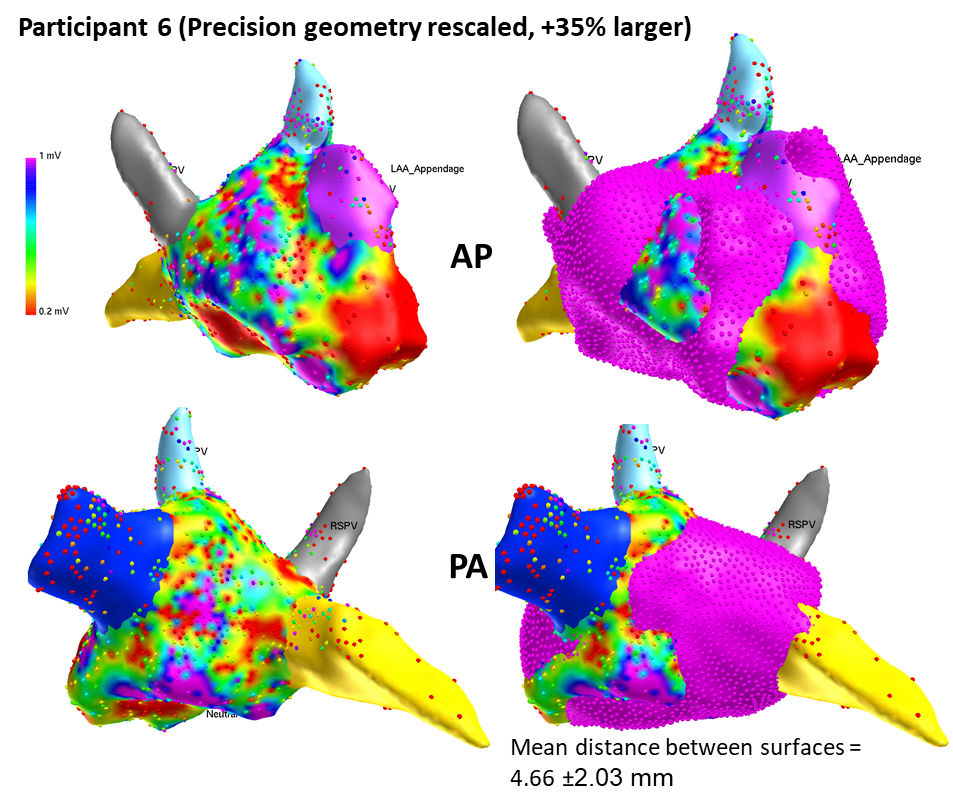


**Figure S4** Anteroposterior (AP) and posteroanterior (PA) views of the scaled Precision left atrial geometry (left 2 images) and the merged Precision and AcQMap geometries (right 2 images).

**Supermap and Conduction Heterogeneity Calculation**

Activation mapping was performed during pacing using the AcQMap “Supermap” algorithm (Acutus Medical) in a subset of 9 patients recruited following the introduction and clinical release of this mapping algorithm. The pacing protocol involved a repeating 4-beat drive train followed by a single extrastimulus at a coupling interval of 20ms longer that the effective refractory period with no rest period, using a pacing output of twice the diastolic threshold. This was conducted at up to 3 sites including the high right atrium, left atrial appendage (both using the ablation catheter positioned at these sites), and the most proximal coronary sinus bipoles positioned within the LA. If AF was induced by the pacing protocol, DCCV was used to restore sinus rhythm but pacing was abandoned if AF was repeatedly induced and only data collected up to that point analysed.

The “Supermap” algorithm allows continuous data collection across multiple paced cycles. The AcQMap basket was roved throughout the chamber for a minimum duration of 3 minutes to ensure full and even coverage of the entire chamber. Each paced beat (drive train and extrastimulus) is identified automatically using the morphology and cycle length of unipolar signals detected with the coronary sinus catheter electrodes and local chamber electrogram data is “binned” according to the corresponding group identified by the coronary sinus signals. This allows simultaneous mapping of both paced cycle lengths and was repeated for each pacing site and within the LA and RA.

Local activation time (LAT) data from each map created was exported and analysed using a custom application programme. The LAT difference between each vertex and every neighbouring vertex at a 5mm radius was calculated and the maximum value assigned to that local point. Values <1ms or >100ms were excluded as these were deemed to suggested non-physiological conduction of either <0.05m/s or >5m/s. When applied to the whole chamber, or specific regions, a histogram of LAT differences could be generated and the median value for that specific region calculated to give the median activation time difference (MAT). The conduction heterogeneity index (CHI) was derived from the difference between the 95^th^ and 5^th^ percentile values divided by the MAT as illustrated in figure S5 and described previously (reference 8 in manuscript). As high frequency LIA zones were found to be stable across maps, these regions were identified on the 1^st^ AF map obtained in each participant and an additional 5mm radius added to this region. The difference in MAT and CHI between each paced cycle length was compared within this region and in the remainder of the chamber.


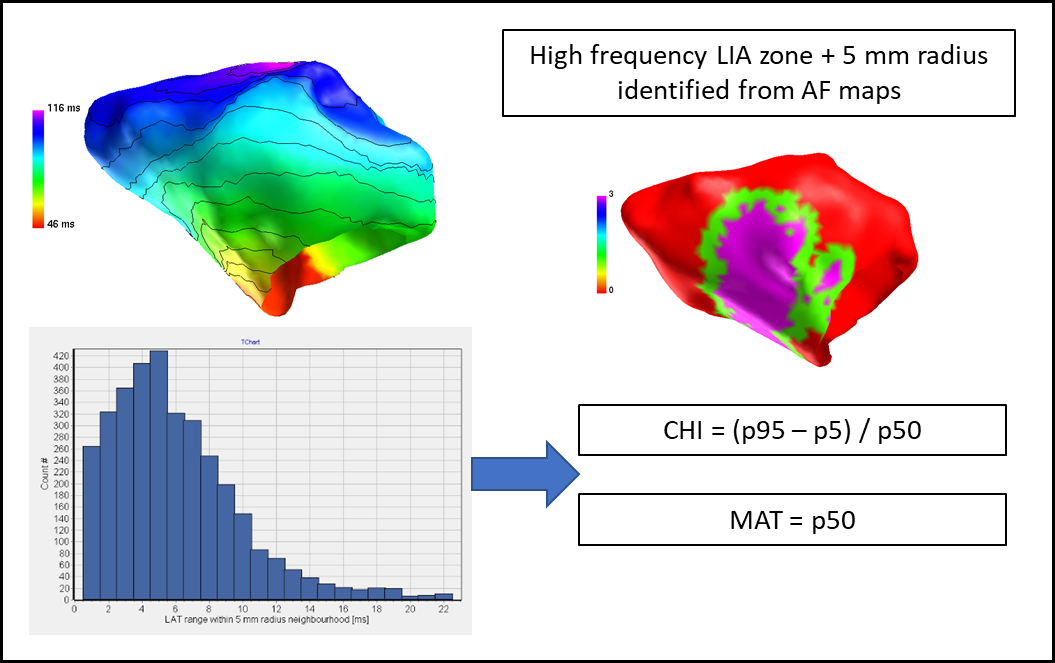


**Figure S5**. Local activation time maps are obtained using supermap during pacing and regions with high frequency LIA are identified during AF. LAT differences at a radius of 5mm from every point on the chamber surface are calculated allowing generation of a histogram for whole region concerned (e.g. LIA or LRA zones). The conduction heterogeneity index (CHI) and median activation time difference (MAT) are calculated from this histogram.

**Supplementary results:**

| **Group** | | **Time to reach kappa 0.8** | **Difference (95% CI)** | **P value** |
| --- | --- | --- | --- | --- |
| **AF classification** | Paroxysmal | 15(4) | 4 (0.6-5.6) | 0.004 |
|  | Persistent | 11(6) |  |  |
| **Chamber** | LA | 12.4(4.3) | 0.1 (-1.7 – 1.5) | 0.898 |
|  | RA | 12.5(4.8) |  |  |
| **Ablation stage** | prePVI | 13.0(4.9) | 1.5 (-0.3-3.1) | 0.099 |
|  | postPVI | 11.5(3.5) |  |  |
| **Antiarrhythmics** | On amiodarone | 10(6) | 3 (0.1-3.6) | 0.007 |
|  | Off amiodarone | 13(6) |  |  |

**Table S1** Differences in time to reach a kappa value of 0.8 between zones of high frequency LIA compared to 30-seconds. LIA, localised irregular activation; LA, left atrium; RA, right atrium; PVI, pulmonary vein isolation

| **Group** | | **Time to reach kappa 0.8** | **Difference (95% CI)** | **P value** |
| --- | --- | --- | --- | --- |
| **AF classification** | Paroxysmal | 19.8 (5.4) | 1.5 (-4.3 – 1.4) | 0.314 |
|  | Persistent | 21.3 (5.4) |  |  |
| **Chamber** | LA | 20.7 (4.9) | 0.7 (-2.6 – 1.2) | 0.491 |
|  | RA | 21.3 (5.8) |  |  |
| **Ablation stage** | Pre-PVI | 21.1 (4.6) | 0.2 (-1.8 – 2.3) | 0.822 |
|  | Post-PVI | 20.9 (6.7) |  |  |
| **Antiarrhythmics** | On amiodarone | 21.1 (6.0) | 0.0 (-2.0-2.0) | 0.985 |
|  | Off amiodarone | 21.1 (5.1) |  |  |

**Table S2** Differences in time to reach a kappa value of 0.8 between zones of high frequency LRA compared to 30-seconds. LRA, localised rotational activation; LA, left atrium; RA, right atrium; PVI, pulmonary vein isolation

| **Group** | | **Time to reach kappa 0.8** | **Difference (95% CI)** | **P value** |
| --- | --- | --- | --- | --- |
| **AF classification** | Paroxysmal | 19.9 (3.4) | 1.6 (-0.8 – 3.4) | 0.186 |
|  | Persistent | 18.3 (4.7) |  |  |
| **Chamber** | LA | 18.6 (4.2) | 0.2 (-1.4 – 1.9) | 0.828 |
|  | RA | 18.4 (4.9) |  |  |
| **Ablation stage** | Pre-PVI | 19.1 (3.7) | 1.9 (0.3 – 3.6) | 0.023 |
|  | Post-PVI | 17.2 (5.6) |  |  |
| **Antiarrhythmics** | On amiodarone | 18.6 (4.8) | 0.2 (-1.5 – 1.9) | 0.835 |
|  | Off amiodarone | 18.4 (4.4) |  |  |

**Table S3** Differences in time to reach a kappa value of 0.8 between zones of high frequency FF compared to 30-seconds. FF, focal firing; LA, left atrium; RA, right atrium; PVI, pulmonary vein isolation.

**
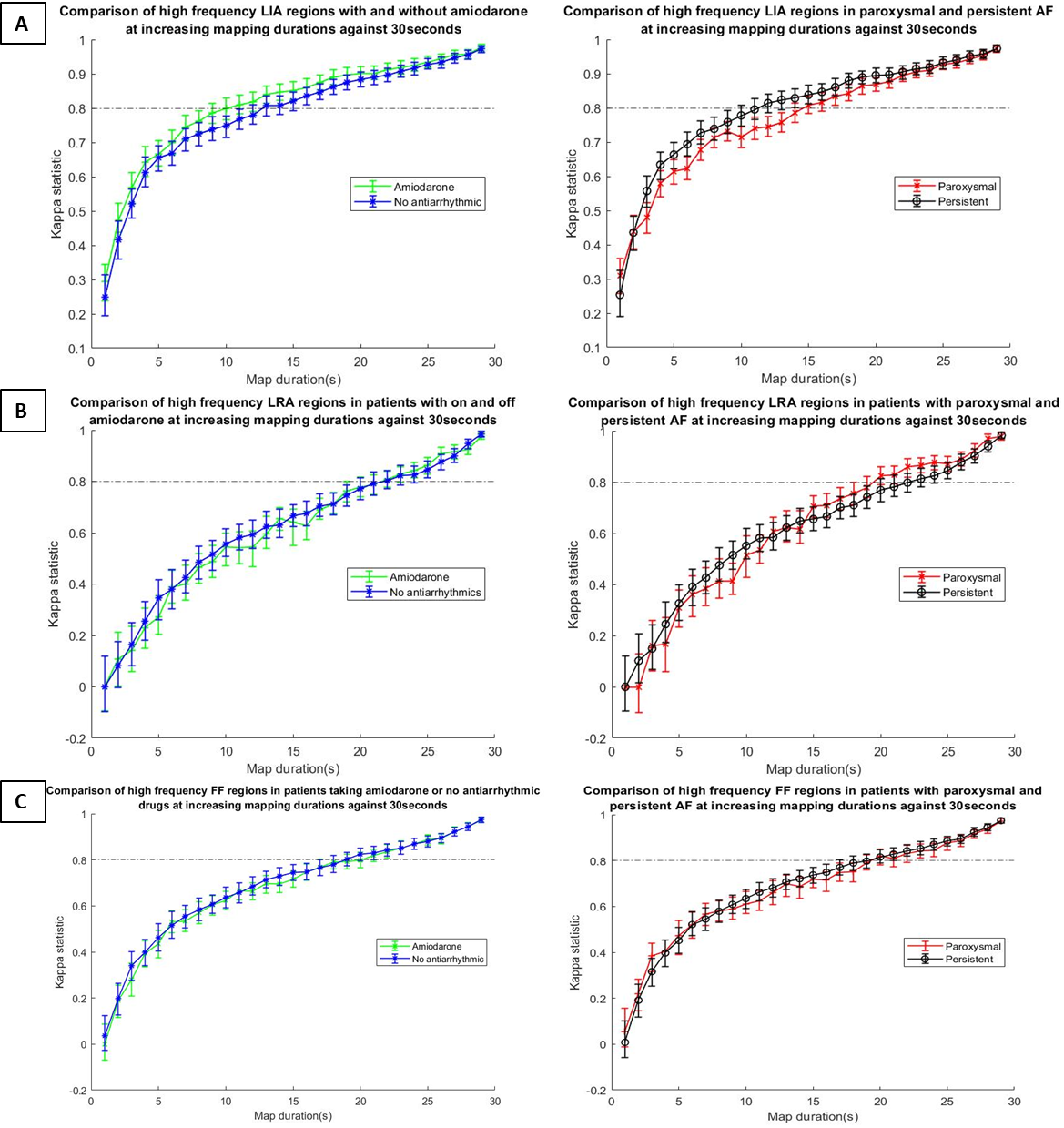
Figure S6** Comparison between patients on or off amiodarone and with paroxysmal or persistent AF on the agreement between zones with high frequency LIA (A), LRA (B) and FF (C) at incremental mapping durations against 30s.

**Figure S7** Comparison between patients before and after pulmonary vein isolation and in the left compared to the right atrium on the agreement between zones with high frequency LIA (A), LRA (B) and FF (C) at incremental mapping durations against 30s.
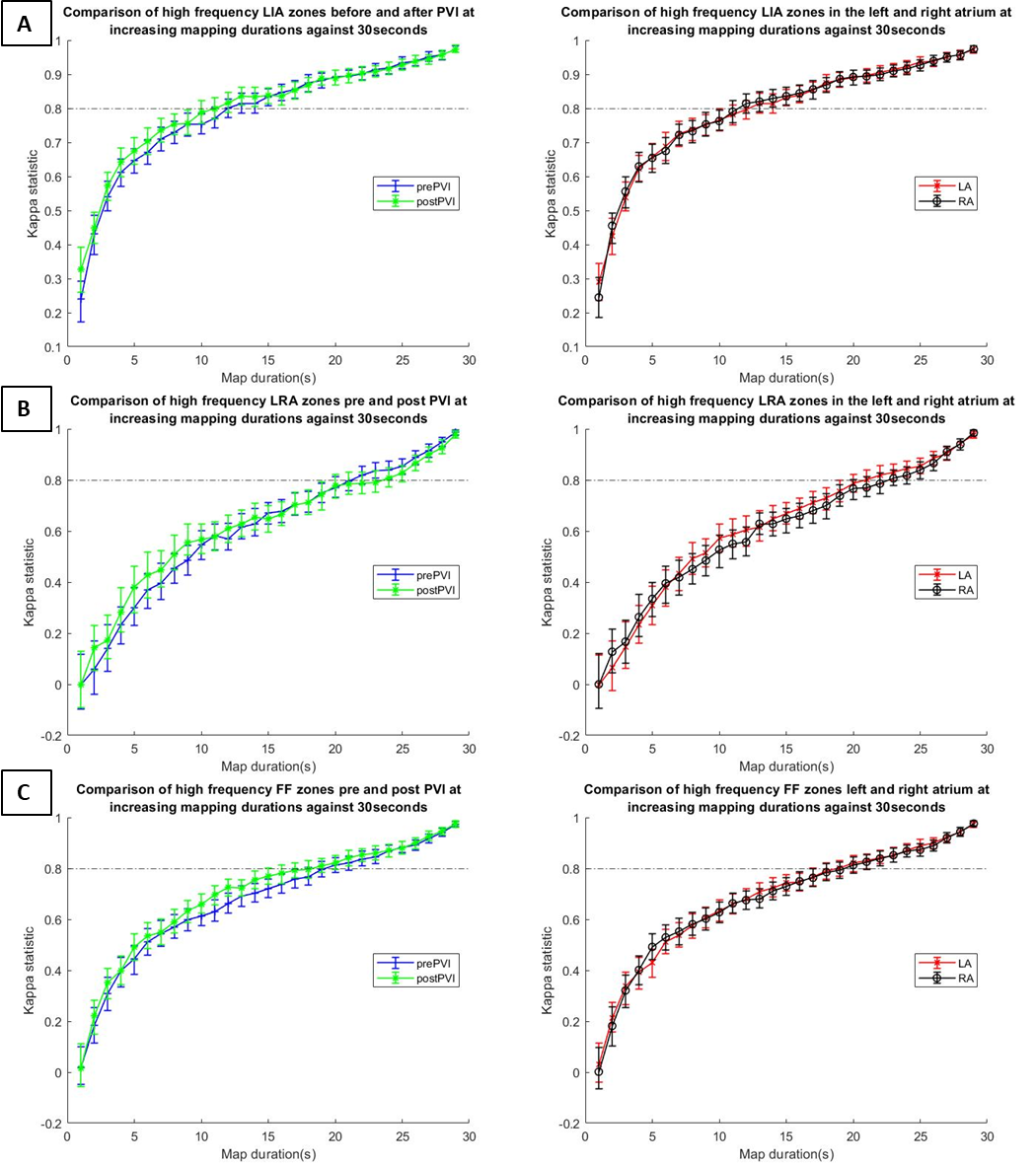


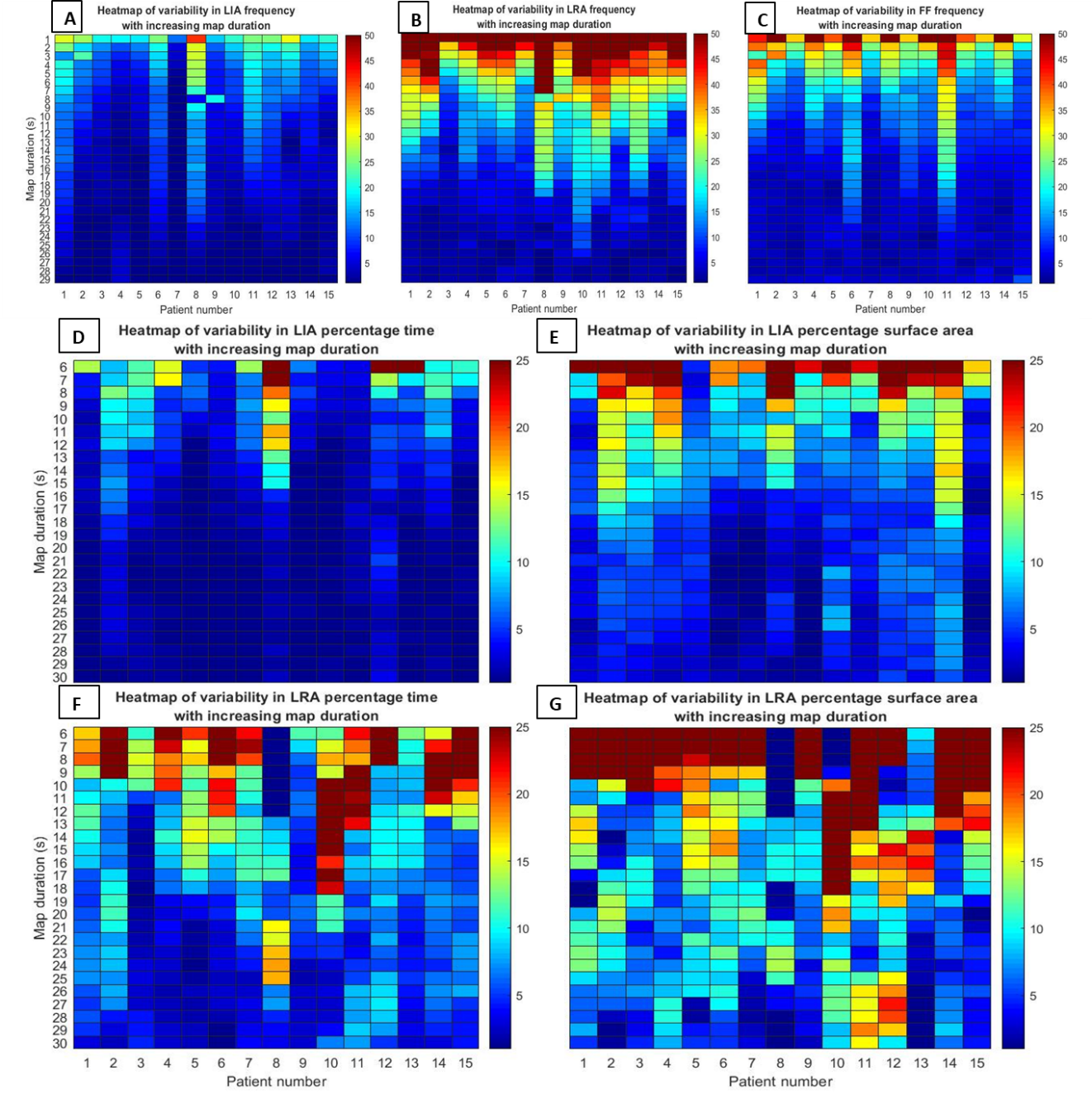
**Figure S8** Heatmaps showing the variability in frequency of LIA, LRA and FF (A-C) in a subset of 15 patients studied with colours representing the percentage change in each pattern at incremental recording duration. The variability of patterns within the highest frequency regions identified using the 30% cut off are shown as a percentage time and a proportion (%) the atrial surface area over which it occurs for both LIA (D-E) and LRA (F-G).


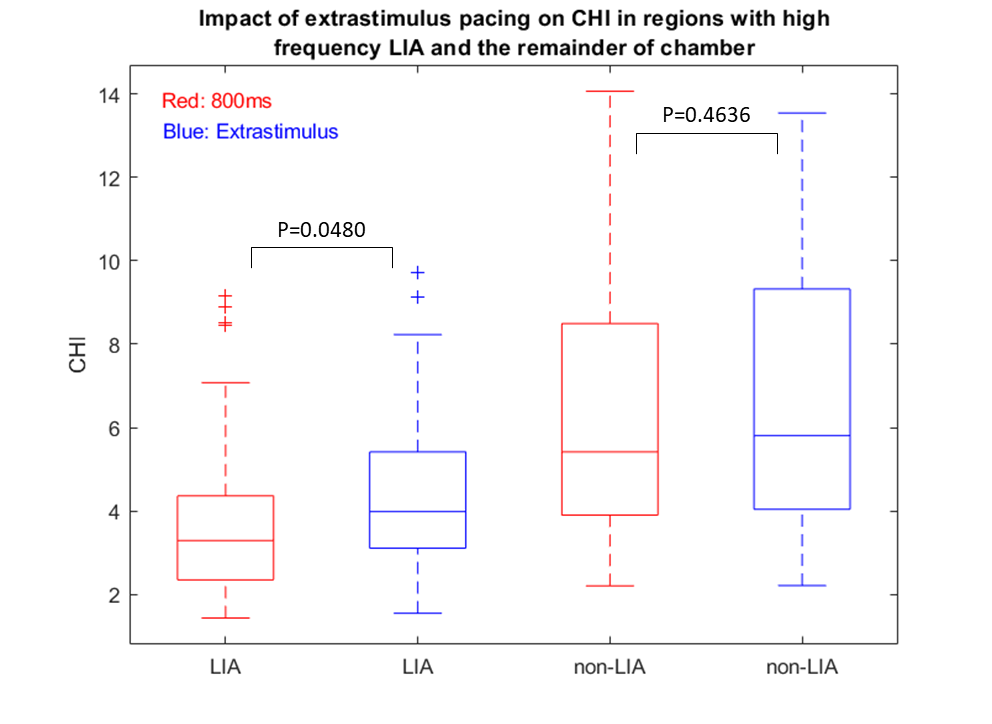


**Figure S9.** Boxplot of the effect of pacing cycle length on CHI in regions with high frequency LIA and the remainder of the chamber.
